# The Alterations in Mitochondrial Dynamics Following Cerebral Ischemia/Reperfusion Injury

**DOI:** 10.3390/antiox10091384

**Published:** 2021-08-30

**Authors:** Jirapong Vongsfak, Wasana Pratchayasakul, Nattayaporn Apaijai, Tanat Vaniyapong, Nipon Chattipakorn, Siriporn C. Chattipakorn

**Affiliations:** 1 Neurophysiology Unit, Cardiac Electrophysiology Research and Training Center, Faculty of Medicine, Chiang Mai University, Chiang Mai 50200, Thailand; jvongsfak@gmail.com (J.V.); wpratcha@med.cmu.ac.th (W.P.); napaijai@gmail.com (N.A.); nchattip@gmail.com (N.C.); 2 Neurosurgery Unit, Department of Surgery, Faculty of Medicine, Chiang Mai University, Chiang Mai 50200, Thailand; tanat@neurosurgerycmu.com; 3 Cardiac Electrophysiology Unit, Department of Physiology, Faculty of Medicine, Chiang Mai University, Chiang Mai 50200, Thailand; 4 Center of Excellence in Cardiac Electrophysiology, Chiang Mai University, Chiang Mai 50200, Thailand; 5 Department of Oral Biology and Diagnostic Sciences, Faculty of Dentistry, Chiang Mai University, Chiang Mai 50200, Thailand

**Keywords:** mitochondria, fission, fusion, ischemia, reperfusion

## Abstract

Cerebral ischemia results in a poor oxygen supply and cerebral infarction. Reperfusion to the ischemic area is the best therapeutic approach. Although reperfusion after ischemia has beneficial effects, it also causes ischemia/reperfusion (I/R) injury. Increases in oxidative stress, mitochondrial dysfunction, and cell death in the brain, resulting in brain infarction, have also been observed following cerebral I/R injury. Mitochondria are dynamic organelles, including mitochondrial fusion and fission. Both processes are essential for mitochondrial homeostasis and cell survival. Several studies demonstrated that an imbalance in mitochondrial dynamics after cerebral ischemia, with or without reperfusion injury, plays an important role in the regulation of cell survival and infarct area size. Mitochondrial dysmorphology/dysfunction and inflammatory processes also occur after cerebral ischemia. Knowledge surrounding the mechanisms involved in the imbalance in mitochondrial dynamics following cerebral ischemia with or without reperfusion injury would help in the prevention or treatment of the adverse effects of cerebral injury. Therefore, this review aims to summarize and discuss the roles of mitochondrial dynamics, mitochondrial function, and inflammatory processes in cerebral ischemia with or without reperfusion injury from in vitro and in vivo studies. Any contradictory findings are incorporated and discussed.

## 1. Introduction

Stroke is a leading cause of mortality and disability worldwide. Approximately 85% of strokes are termed cerebral ischemia, and 15% are termed cerebral hemorrhage [1]. Cerebral ischemia is caused by the occlusion of cerebral arteries, resulting in a lack of blood supply and, hence, a loss of glucose/oxygen to all cells in the brain. Blood deprivation to the brain disturbs brain cell homeostasis and triggers pathophysiological conditions in the brain, including an increase in oxidative stress, inflammation, excitotoxicity, and culminating in brain cell death. A therapeutic approach for cerebral ischemia can be via thrombolysis in order to restore the blood supply into the brain. However, rapid reperfusion can result in further injury to the brain area, a condition known as ischemia/ reperfusion injury. Several studies report that the pathophysiological conditions following stroke or permanent and transient cerebral ischemia are associated with mitochondrial dysfunction and impaired mitochondrial dynamics in the brain [2,3,4]. Other neurological diseases are associated with mitochondrial dynamic changes. Charcot–Marie–Tooth disease, Huntington’s disease, Parkinson’s disease, and neurodegenerative diseases are related to the disruption of fusion, fission, mitophagy, and biogenesis processes in mitochondria [5,6,7]. These impaired mitochondrial processes cause mitochondrial dysfunction and consequently cause neuronal dysfunction.

Studies from the past twenty years have focused on the effects of alterations in mitochondrial dynamics and their interventions to balance mitochondrial dynamics in the conditions of permanent and transient cerebral ischemia in both in vitro and in vivo models. The models of permanent and transient cerebral ischemia in in vitro studies have been those using oxygen-glucose deprivation (OGD) or glutamate-induced neuronal toxicity, and reperfusion with reoxygenation. The study models for in vivo studies have been associated with global cerebral ischemia or transient middle cerebral artery occlusion (MCAO), and reperfusion after cerebral ischemia. Several studies have demonstrated that the imbalance of mitochondrial dynamics occurs in permanent and transient cerebral ischemia in order to protect further damage to the brain [8,9,10]. However, controversial findings about the possibly beneficial effects of imbalanced mitochondrial dynamics on permanent and transient cerebral ischemia have been reported [11,12,13]. Therefore, the aim of this review is to summarize the evidence from in vitro and in vivo studies associated with the effects of mitochondrial dynamics on the brain with permanent and transient cerebral ischemia and identify any contradictory findings. Information about the alteration in mitochondrial dynamics during permanent and transient cerebral ischemia may be an important gateway to understanding the role of mitochondrial dynamics during a stroke and after conventional therapeutic approaches.

## 2. Mitochondrial Dynamics in the Brain under Normal Physiological Conditions

Mitochondria are intracellular organelles whose functions are important for cell survival. The principal role of mitochondria is to generate intracellular energy production (ATP) via the oxidative phosphorylation pathway [2]. Mitochondria are also essential for calcium regulation and the maintenance of membrane potential in cells. Under normal conditions, mitochondria are organelles with dynamics that consist of mitochondrial fusion and mitochondrial fission. The balance between these two processes is essential for mitochondrial homeostasis and cell survival [4]. Mitochondrial fission is the process for the division of mitochondria. This process enables mitochondria to segregate dysfunctional mitochondria which contain damaged protein, mutated DNA, or destabilized membranes [4]. Mitochondrial fission is mediated by dynamic related protein 1 (Drp1), which is essential for mitochondrial division [14]. Mitochondrial fission is also associated with the mitophagy process, which is the process that separates and removes damaged mitochondria to maintain intracellular homeostasis in the process of mitochondrial quality control [15,16]. Several studies reported that Drp1-mediated mitochondrial fission could modulate mitophagy via the Pink1-Parkin signaling pathway or the mitophagy receptors Nip3-like protein X (NIX) and Bcl2/adenovirus E1B 19-kDa-interacting protein-3 (Bnip3), consequently caused the removing damaged mitochondria by autophagosome engulfment [16,17,18]. Furthermore, mitochondrial fission is associated with the mitochondrial biogenesis, which is the process that increases mitochondrial numbers. Several studies reported that peroxisome proliferator-activated receptor-gamma coactivator-1α (PGC-1α), which is a transcriptional coactivator that regulates mitochondrial biogenesis, could regulate Drp1 protein expression and its phosphorylation [19,20].

Mitochondrial fission plays an important role during synapse formation in dendritic spines [21]. Hippocampal neurons transfected with dominant-negative Drp1 showed a depletion of mitochondria in their dendrites, along with a reduction of dendritic spine density [22]. Therefore, mitochondrial fission is necessary for mitochondrial distribution to the dendrites and facilitates the synapses of the neurons. Mitochondrial fission process requires Drp1 translocation from cytosol to the outer membrane of mitochondria where mitochondrial fission receptor and proteins reside at the constriction site, including mitochondrial fission 1 protein (Fis 1), mitochondrial fission factor (MFF), and MiD49/51 [23]. Then, the GTP is hydrolyzed to enhance endoplasmic reticulum-mediated mitochondrial constriction [23]. At the inner mitochondrial membrane, mitochondrial fission occurs in a mitochondrial calcium-dependent manner together with an increased short form of optic atrophy 1 (s-OPA-1). s-OPA1 is involved in untethering the OMM from the IMM during fission [23]. There is a study suggesting that constriction of inner mitochondrial compartment is a priming event for mitochondrial fission process [24].

**Figure 1:**
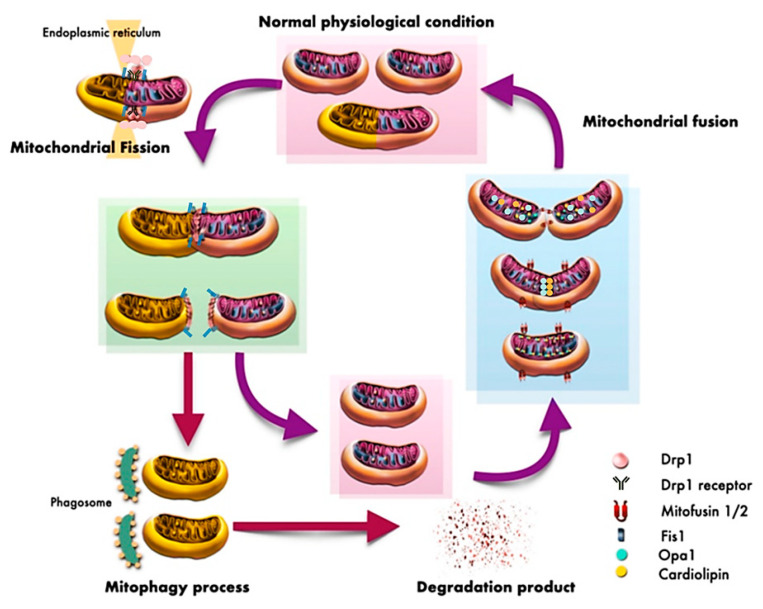
Under the physiological condition, mitochondrial dynamic is a process for the balance between fission and fusion to provide cellular homeostasis and cell survival. Mitochondrial fission is the process for the division of mitochondria. Mitochondrial fission enables mitochondria to segregate dysfunctional mitochondria. Mitochondrial fission is mediated by Drp1 and Fis1, which are essential for mitochondrial division. In addition, mitochondrial fission is associated with the mitophagy process, which is the process that separates and removes damaged mitochondria by a phagosome. Mitochondrial fusion involves the processes of sharing the mitochondrial matrix or metabolites. Mfn1, Mfn2, and OPA1 play roles in mitochondrial fusion. The long form of OPA-1 interacts with cardiolipin of the adjacent mitochondria to promote inner mitochondrial fusion. Drp1, dynamin-related protein-1; Fis1, mitochondrial fission 1 protein; Mfn1, mitofusin1; Mfn2, mitofusin2; OPA1, optic atrophy protein1.

Mitochondrial fusion is the process for the merging of mitochondria. This process involves the processes of sharing the mitochondrial matrix or metabolites such as proteins, mitochondrial DNA, or membrane components where the electron transport chain occurs [4]. Three different GTPases, including mitofusin 1 (Mfn1), mitofusin 2 (Mfn2), and optic atrophy 1 (OPA1), play roles in mitochondrial fusion. Mfn1 and Mfn2 are located on the outer mitochondrial membrane, while OPA1 mediates the process of mitochondrial fusion at the inner membrane of the mitochondria [14]. Regarding the two forms of OPA1, we mentioned earlier that the short form of OPA-1 or s-OPA1 mediates inner mitochondrial membrane fission. By contrast, the long form of OPA-1 (L-OPA1) interacts with cardiolipin (a unique inner mitochondrial membrane protein) of the adjacent mitochondrion to promote inner mitochondrial fusion. The summary of mitochondrial dynamics under physiological conditions is shown in Figure 1.

For the evaluation of mitochondrial dynamics processes, there are several methods to evaluate and monitor mitochondrial dynamics processes via the detection of mitochondrial dynamic proteins, mitochondrial morphology, mitochondrial cristae integrity, or mitochondrial size. Mitochondrial dynamic proteins such as Drp1, pDrp1, Mfn2, and OPA1 were determined by Western blot analysis [25,26,27,28]. The evaluation of mitochondrial morphology, mitochondrial cristae integrity, and mitochondrial size was detected by immunohistochemistry or immunofluorescence and was visualized by confocal microscopy or transmission electron microscopy [8,25,26].

## 3. Mitochondrial Dynamics in the Brain under Pathological Conditions

**Figure 2:**
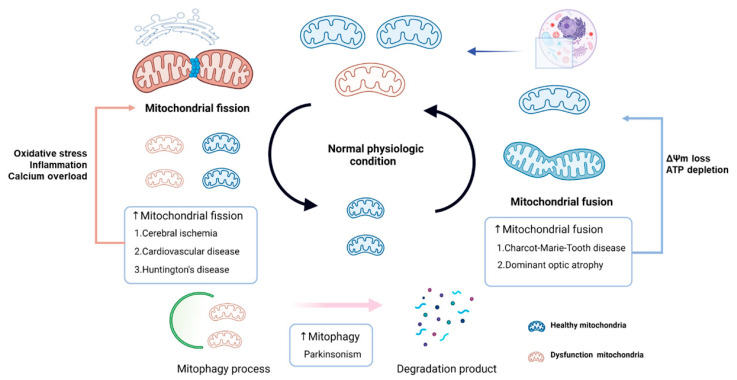
A schematic diagram representing the alteration of mitochondrial dynamic balance during pathology. Several diseases are related to the disruption of fusion, fission, and mitophagy in mitochondria. Cerebral ischemia, cardiovascular disease, and Huntington’s disease increased mitochondrial fission via increased oxidative stress, inflammation, and calcium overload. Charcot–Marie–Tooth disease and dominant optic atrophy increased mitochondria fusion by stimulated mitochondrial membrane depolarization and ATP depletion. Parkinson’s disease also increased mitophagy. ATP, adenosine triphosphate; ΔΨm, mitochondrial membrane potential. (This figure was created with BioRender.com).

Most of the evidence from pathological conditions, including cardiovascular diseases, metabolic disorders, and neurological diseases, has demonstrated an increase in mitochondrial fission as indicated by increased expression of Drp1 and a decrease in mitochondrial fusion as indicated by the reduction of OPA1 and Mfn2 expression, causing an imbalance in the dynamics of the mitochondria [11,29], resulting in mitochondrial fragmentation [25]. Some evidence demonstrated that an imbalance in the mitochondrial dynamics plays an essential role in the cerebral ischemic condition [2]. Both in vitro and in vivo studies showed that the inhibition of mitochondrial fission by the Drp1 inhibitor or siRNA had beneficial effects on cerebral ischemia [8,9,28], but a previous study had contradictory findings [11]. Cerebral ischemia has also been shown to result in the alterations in mitochondrial dynamics, leading to an inability of mitochondria to produce energy, an increase in cell death pathways, mitochondrial dysfunction, mitochondrial dysmorphology, and impaired mitophagy as indicated by deteriorating Parkin or cAMP/PKA/CREB signaling pathways [25,26]. A previous study showed that Parkin overexpression protected mitochondrial dysfunction in neuroblastoma cells following oxygen-glucose deprivation/reoxygenation via increased Drp1 degradation [30]. All of these findings suggest that the important underlying mechanisms behind cerebral ischemia are associated with imbalanced mitochondrial dynamics. Therefore, the effect of permanent and transient cerebral ischemia on the alteration in mitochondrial dynamics and mitochondrial dysfunction need consideration and are summarized and discussed in the following section. The summary of mitochondrial dynamics under pathological conditions is shown in Figure 2.

## 4. Evidence Pertinent to the Alteration in Mitochondrial Dynamics Following Oxygen-Glucose Deprivation/Reoxygenation: In Vitro Studies

An in vitro model of oxygen glucose deprivation (OGD), with or without reoxygenation, was used to determine the alteration in mitochondrial dynamics following ischemia and reoxygenation. Regarding the OGD condition, four in vitro studies investigated the alterations in mitochondrial dynamics. The impact of 60 min of OGD in a primary culture of mouse optic nerves resulted in an imbalance in mitochondrial dynamics, as indicated by decreased cytosolic Drp1 expression [13]. The study of 45 min of OGD caused mitochondrial fragmentation in a hippocampal neuronal culture [25]. Three hours of hypoxia caused decreased pDrp1 (ser637) expression in primary astrocytes, resulting in increased mitochondrial fragmentation, increased mitochondrial autophagy, and decreased mitochondrial ATP production [31]. All findings suggested that an increase in mitochondrial fission occurred in ischemic conditions. By contrast, 3 h of OGD decreased Drp1, pDrp1 (ser616), and Mfn2 expression in the primary cortical neuronal culture [32]. In addition, decreased normal mitochondria and increased poorly and swollen mitochondria were observed in the hypoxic neurons [32]. Thus, in the cortical neurons, mitochondrial fission may occur in neurons in a Drp1-independent manner possibly through the activation of Fis1. Drp1 and Fis1 take a different action to induce mitochondrial fission and apoptosis as Fis1 mediated Bax translocation to the outer mitochondrial membrane, whereas Drp1 regulated a release of cytochrome c [33].

After a reoxygenation/reperfusion period, mitochondrial fission and fragmentation were increased in neuronal cultures [25,26,30,31]. Mitochondrial fusion was significantly decreased in hippocampal neurons following 45 min of OGD and 5 h of reoxygenation, as indicated by increased s-OPA1 levels [25]. Similarly, one hour of OGD followed by 24 h of reoxygenation in both cortical and hippocampal neurons caused an increase in OPA1 mitochondrial translocation, increased s-OPA1 levels in the cytosol, and a breakdown of OPA1 oligomers in the mitochondria [26]. Therefore, the disruption of the OPA1 oligomer during reperfusion contributed to an increase in mitochondrial fragmentation and neuronal death. All of these findings suggest that mitochondrial fission was increased, and mitochondrial fusion was decreased after OGD and a reoxygenation period. However, 3 h of OGD with 0.5–24 h of reoxygenation in primary cortical neurons showed the reduction of Drp1, pDrp1 (ser616), and Mfn2 expression with the increment of Mfn1 expression [32]. Increases in swollen mitochondria, voltage-dependent anion-selective channel (VDAC), complex V protein, and mitochondrial DNA (mtDNA) were observed in the OGD and a reoxygenation condition [32]. Evidence pertinent to the alterations in mitochondrial dynamics following oxygen-glucose deprivation/reoxygenation is summarized in Table 1. It has been shown that apoptosis is related to mitochondrial fragmentation [25]. Therefore, we also summarized the changes in apoptotic markers, which were related to alterations in mitochondrial dynamics following OGD/reoxygenation. After 45 min of OGD and 5 h of reoxygenation in a hippocampal neuronal culture, apoptosis was not detected during OGD but was observed after reoxygenation, as indicated by increased caspase 3/7 activation [25]. Cytochrome c is a soluble protein that is located on the inner membrane of mitochondria and is associated with the initiation of the cell death cascade. After 1 h of OGD followed by 24 h of reoxygenation, cytochrome c levels were significantly increased in both cortical and hippocampal neurons [26]. Furthermore, the decrement of neuronal viability and astrocytic extensions were observed after OGD and reoxygenation period [31]. All of these findings suggest that OGD with reoxygenation triggers an imbalance in mitochondrial dynamics, resulting in neuronal cell death.

## 5. Evidence for the Alterations in Mitochondrial Dynamics Following Cerebral Ischemia/Reperfusion Injury: In Vivo Studies

In in vivo studies, middle cerebral artery occlusion (MCAO) and global ischemia/reperfusion models were used to determine the alterations in mitochondrial dynamics following cerebral ischemia/reperfusion injury. During the ischemic period, 12 and 24 h after MCAO in C576BL/6 mice, attenuation of brain Mfn2 levels were shown [34]. At 15 min of global ischemia in Wistar rats, an increase in brain Mfn2 translocation from mitochondria to cytoplasm was observed [35]. All of these findings suggest that mitochondrial fusion decreased after the ischemic period in in vivo studies.

With regard to cerebral ischemia/reperfusion, 8 min of global ischemia with 4–24 h of reperfusion increased OPA1 release in the brain of Sprague-Dawley rats [25], whereas 15 min of global ischemia with 1–72 h of reperfusion in Wistar rats decreased the expression and translocation of Mfn2 in cortical mitochondria but not in hippocampal mitochondria [35]. Two hours of MCAO with three hours of reperfusion increased the number of small and spherical shaped mitochondria, a phenomenon related to mitochondrial fission in the brain of C576BL/6 mice [36]. All of these findings also showed mitochondrial fragmentation and a loss of normal cristae structure in the brain mitochondria following the cerebral ischemia/reperfusion.

Focusing on the area of infarction in a 90 min transient MCAO and reperfusion in mice model, both Drp1 and OPA1 levels were decreased in the ischemic core of the cerebral infarction, which was due to necrotic brain damage [27]. In the ischemic penumbra area, mitochondrial fission and fusion activities increased continuously, as indicated by an increase in both OPA1 and Drp1 levels 2–18 days postreperfusion [27]. Furthermore, alterations in mitochondrial dynamics were observed in several brain regions following cerebral ischemia/reperfusion. Ten minutes of global ischemia followed by from 2 h to 3 days of reperfusion in transgenic mice with mitochondrial tagging, the rate of mitochondrial fission in the CA1 hippocampus was increased [37]. However, there was an increase in mitochondrial fusion in the CA3 and dentate gyrus areas in the 24 h after reperfusion, and the maximum level of mitochondrial fusion was observed at 3 days after reperfusion [37]. Mitochondrial fission was observed in astrocytes 2 h after reperfusion and was returned to normal after 24 h of reperfusion [37]. This evidence suggests that resistant ischemic neurons can shift the mitochondrial fission to fusion process, particularly in the 24 h following cerebral ischemia/reperfusion.

**Figure 3:**
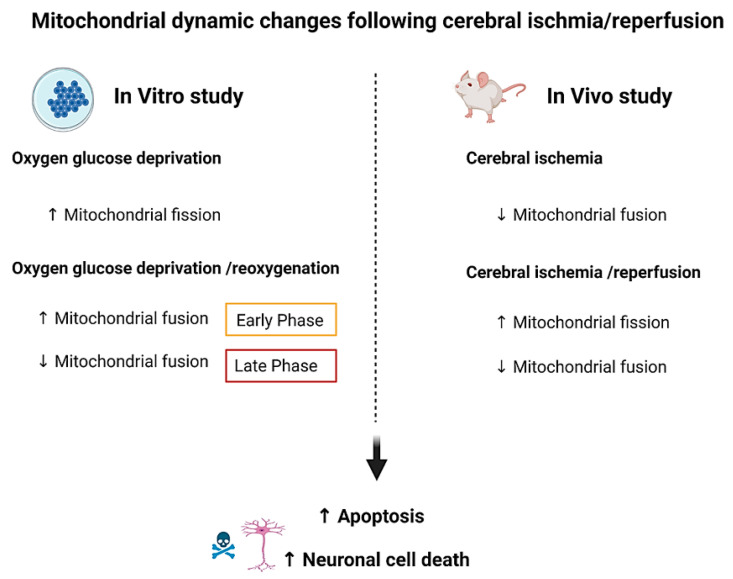
The differences in mitochondrial dynamic alteration after ischemia with or without reperfusion (both in vitro and in vivo studies). In in vitro studies, an increase in mitochondrial fission occurred in OGD (ischemic) condition. Mitochondrial fusion was increased during the early phase of OGD/reoxygenation and decreased after late phase of OGD/reoxygenation. In in vivo studies, a decrease in mitochondrial fusion occurred in cerebral ischemia. An increase in mitochondrial fission and the reduction in mitochondrial fusion were observed after cerebral ischemia/reperfusion. These impaired mitochondrial dynamics caused brain apoptosis and consequently caused neuronal cell death. OGD, oxygen glucose deprivation. (This figure was created with BioRender.com).

The alterations in mitochondrial dynamics after cerebral ischemia/reperfusion in Sprague-Dawley rats were associated with the increase in cytochrome c release and caspase 3/7 activation in global cerebral ischemic neurons [25] and increased voltage-dependent anion channel-1 (VDAC1) expression in the hippocampus of Wistar rats in the following 3–72 h after reperfusion [35]. Furthermore, the mitochondrial recruitment of Drp1 activated the Parkin-mediated mitophagy, leading to an increase in the infarct size in 2 h of MCAO with 22 h of reperfusion in Sprague-Dawley rats [38]. All these findings suggest that fission and fragmentation in mitochondria increased with the reduction in mitochondrial fusion after ischemia/reperfusion, resulting in cell death. Furthermore, the alterations in mitochondrial dynamics in the brain were dissimilar in the ischemic core, ischemic penumbra, and area of infarction. A summary of the evidence pertinent to alterations in mitochondrial dynamics following cerebral ischemia/reperfusion injury in in vivo studies is shown in Table 2. A summary of evidence in the alterations in mitochondrial dynamics following ischemia/reperfusion in in vitro and in vivo studies is summarized in Figure 3.

## 6. Evidence from the Interventions for the Alterations in Mitochondrial Dynamics Following Oxygen-Glucose Deprivation/Reoxygenation: In Vitro Studies

Several therapeutic studies targeted mitochondrial dynamics in association with oxygen-glucose deprivation/reoxygenation, as shown in Table 3. Mitochondrial fission inhibition by either Drp1 siRNA or a Drp1 inhibitor (Mdivi-1) decreased mitochondrial fragmentation, mitochondrial depolarization/membrane potential, mitochondrial swelling, and apoptotic cells in OGD and glutamate-induced neurotoxic models [8,28]. Treatment with Drp1 siRNA 72 h prior to glutamate-induced neurotoxicity increased ATP production and preserved mitochondrial function via decreased mitochondrial swelling and increased mitochondrial calcium buffering capacity in HT-22 hippocampal cells [28]. Drp1 siRNA given 72 h prior to instigation of glutamate-induced neurotoxicity decreased apoptotic processes in HT-22 hippocampal cells [28]. Ninety minutes of OGD with 24 h reoxygenation, the inhibition of Drp-1 decreased Bax in the mitochondrial fraction and decreased cytochrome c release in cytosol, resulting in the reduction of cell death [9]. Knockdown or inhibition of Drp1 decreased mitochondrial morphological changes with an increase in mitochondrial function, leading to attenuation of neurotoxicity following OGD [9]. Furthermore, the administration of the Drp1 inhibitor (Mdivi-1) 40 min prior to OGD suppressed production of reactive oxygen species (ROS) and the apoptotic mitochondrial pathway in hippocampal neurons [39]. The beneficial effects of Mdivi-1 on the increased metabolism of exogenous ATP in astrocytes came through the upregulation of CD39 expression [40]. CD39 is mainly associated with the plasma membrane of astrocytes [41]. The dose-/time-dependent increase in CD39 expression following Mdivi-1 treatment, suggesting that astrocytes are involved in Mdivi-1-induced upregulation of CD39 [40]. Furthermore, CD39 had the ability to degrade ADP to AMP, and Mdivi-1 could regulate cAMP, PKA activity, and the activation of CREB in astrocytes. All of these findings indicate that Mdivi-1 promoted ATP dephosphorylation and adenosine formation via the modulation of CD39 expression through the cAMP/PKA/CREB pathway in astrocytes [40]. Furthermore, pretreatment with Mdivi-1 or Β-hydroxybutyrate, which is a major member of ketone bodies, increased cell viability in SH-SY-5Y cells following OGD via improved the balance of mitochondrial dynamics, mitochondrial function, and decreased ROS production [42]. A beneficial effect of Parkin, which can ubiquitinate Drp1, controlling both biogenesis and degradation of mitochondria via promotion of the mitophagy process [30]. Parkin overexpression protected against mitochondrial dysfunction and apoptosis in neuroblastoma cells following OGD with 4–12 h reoxygenation. This was mediated via increased Drp1 degradation as well as decreased mitochondrial dysfunction [30]. All of these findings suggest that the inhibition of mitochondrial fission by either a Drp1 inhibitor (Mdivi-1), Drp1 siRNA, or Parkin overexpression reduced mitochondrial fragmentation, mitochondrial dysfunction, ROS production, and consequently decreased apoptotic proteins. The decrement of these apoptotic proteins represented a decrease in apoptotic process, possibly resulting in an increase in cell viability under OGD- or glutamate-induced neurotoxic models.

Although several studies have shown the beneficial effects of the inhibition of mitochondrial fission, the negative effect of the inhibition of Drp-1 was observed. Prostaglandin J2 (PJ-2), which is the inhibitor of GPTase activity of Drp1, but not Mdivi-1, increased Drp-1 expression in primary cortical neuronal culture of 3 h OGD followed by 24 h reoxygenation [32]. However, both PJ-2 and Mdivi-1 did not affect mitochondrial morphology and neuronal viability in this study [32]. The explanation of different results from other studies may be due to different models and the methods of cell viability measurement [32]. The application of Drp-1 siRNA 48 h prior to OGD inhibited Drp1 as well as increased LC3-II accumulation and increased the level of LDH, cytochrome c, and ROS in CA1 and CA3 hippocampal neurons [11,12], indicating that inhibiting Drp1 resulted in increased neuronal death, infarct volume, and neurological deficit. The possible explanation of this controversial evidence could be (1) the differences in study model as Zuo and colleagues investigated the effect of inhibiting mitochondrial fission on cell survival and oxidative stress in the case of OGD without reoxygenation, and (2) this study only focused on the early period (within 24 h) after OGD. In addition, the report by Zuo and colleagues suggested that mitochondrial fission occurred in the early phase after cerebral ischemia as it may be essential to instigate the mitophagy process in order to get rid of damaged mitochondria.

Intervention in mitochondrial fusion was focused on the regulation of Mfn2. The upregulation of Mfn2 attenuated hypoxia-induced neuronal apoptosis in CoCl2-induced neuronal toxicity via a decrease in the loss of MMP, decreased phospho-ERK, and an increased Bcl-2/Bax ratio [34]. However, the downregulation of Mfn2 increased apoptosis in this model [34]. All of these findings suggest that inhibiting mitochondrial fission after the onset of OGD with or without reoxygenation had the benefits of decreasing mitochondrial fission, ROS release, and neuronal death. By contrast, the effect of using the mitochondrial fission inhibitor before the onset of OGD or OGD with reoxygenation on brain function is still inconclusive. For mitochondrial fusion, the upregulation of mitochondrial fusion had positive effects via the attenuation of neuronal death after CoCl2-induction.

## 7. Evidence in the Interventions for the Alterations in Mitochondrial Dynamics Following Cerebral Ischemia/Reperfusion: In Vivo Studies

There are eleven therapeutic studies which targeted alterations in mitochondrial dynamics following cerebral ischemia/reperfusion injury, as shown in Table 4. The administration of a Drp1 inhibitor (Mdivi-1) after transient MCAO (tMCAO) with reperfusion decreased brain mitochondrial fragmentation, apoptosis, and brain infarct volume, which resulted in a decrease in neurological deficit [9]. Furthermore, the protective effect of the Drp1 inhibitor in tMCAO and global ischemic models was related to increase cell survival [43] and decreased infarct volume [8]. Similarly, the administration of Mdivi-1 prior to cerebral ischemia or immediately after reperfusion had neuroprotective effects and decreased neurological deficits [40]. The inhibition of Drp-1 with Mdivi-1 after cardiac arrest decreased cytochrome c release in the brain of cardiac arrest and return of spontaneous circulation (ROSC) model [44]. All of these findings imply the protective and beneficial effects of the Drp1 inhibitor on the attenuation of mitochondrial fragmentation, mitochondrial dysfunction, and consequently a decrease in apoptotic cell death, resulting in the reduction of infarct volume and neurological deficits following cerebral ischemia/reperfusion injury.

The deletion of A-kinase anchoring protein 1 (AKAP-1), an endogenous Drp1 inhibitor, increased Drp-1 localization in mitochondria and increased small mitochondria, finally leading to an increase in infarct volume after MCAO with reperfusion [45]. Despite the benefits of the administration of the Drp1 inhibitor before cerebral ischemia, Zuo and colleagues showed that it led to a deterioration in neurological function in permanent MCAO (pMCAO) via increased infarct volume, ROS generation, and apoptosis [11]. The differences in those results caused by the mitochondrial fission inhibitor in Zuo’s study from other similar pieces of research [8,40,43] might be due to: (1) the different models of cerebral ischemia used and (2) the differences in the period of investigation after ischemia. Zuo and colleagues used pMCAO without reperfusion as the model and time point of investigation was only within 24 h after ischemia. However, other studies [8,40,43] which investigated the impact of the Drp1 inhibitor showed beneficial effects in a model of cerebral ischemia/reperfusion. Most of those studies observed beneficial effects at 24 h and 10 days after reperfusion. Zuo and colleagues also showed that ischemia preconditioning (IPC) in a model of 10-min global cerebral ischemia increased neuronal resistance from cerebral ischemia via no change in Drp1 expression in the CA1 hippocampus, increased translocation of Drp1 in the CA3 hippocampus, and increased COX4 and LC3 expression. The administration of the Drp-1 inhibitor in a model of global ischemic brain with IPC impaired brain mitophagy, resulting in increased neuronal damage in the CA3 hippocampus after cerebral ischemia [12]. All of these findings implied IPC in cerebral ischemia had beneficial effects on the brain via the regulation of Drp1, and Drp-1-mediated mitophagy had neuroprotective effects against cerebral ischemia by preventing mitochondrial dysfunction.

Previous studies showed an association between major mitogen-activated protein kinases (MAPK), such as P38, and mitochondrial dynamics in models of cerebral ischemia. P38 MAPK is a cell signaling pathway involved in cellular stress, cell death, apoptosis, or autophagy after cerebral ischemia [46]. The inhibition of P38 MAPKs before tMCAO led to a decrease in brain mitochondrial fission, mitophagy, and infarct size with increased cell survival, leading to decreased neurological deficit [46]. It has been shown that Drp1 is a substrate of P38 MAPK [47]; thus, the inhibition of P38 MAPK directly inhibited Drp1-mediated apoptosis in tMCAO. Moreover, the protective effect of exercise on mitochondrial dynamics was observed in a model of 90 min of tMCAO with 3 h of reperfusion. Two weeks of exercise before ischemic onset increased mitochondrial fusion, improved mitochondrial function, and decreased brain edema [48].

**Table 4:** Evidence in the interventions for the alterations in mitochondrial dynamics following cerebral ischemia/reperfusion: in vivo studies.

Study Model	Injury Model	Intervention	Major Findings					
			Changes in Mitochondrial Dynamics	Mitochondrial Function/Morphology	Cell Survival	Other	Interpretation	Reference
Male C57BL/6 Mice	45 min tMCAO/72 h reperfusion	10 or 20 mg/kg Mdivi-1 after tMCAO	↓ Mitochondrial fragmentation	-	↓ Infarct volume ↓ Cyto c release ↓Bax ↓ Apoptotic cell	↓ Neuronal deficit	Drp-1 inhibitor decreased infarct volume and neuronal death in MCAO model	[9]
Adult male Wister rats	2 h MCAO/24 h reperfusion	0.24, 1.2 mg/kg Mdivi-1 15 min before MCAO	1.2 mg/kg Mdivi-1 ↓ Drp1 Expression	-	1.2 mg/kg Mdivi-1 ↓ Apoptotic cells ↓ Cyto c release	-	Drp-1 inhibitor inhibited mitochondrial fission and neuronal cell death in cerebral I/R	[43]
Male C57BL/6 mice	45 min tMCAO/24 h reperfusion	1, 3 mg/kg Mdivi-1 before MCAO	-	-	↓ Infarct volume	-	Drp-1 inhibitor had neuroprotective effects following I/R	[8]
C57BL/6 mice	45 min MCAO/10 days reperfusion	10, 20 mg/kg Mdivi-1 4 h before MCAO or immediately after reperfusion and every 12 h for 10 days	-	-	Pretreatment ↓ Infarct volume After MCAO ↓ Infarct volume ↑ Extracellular adenosine, ATP ↑ CD39 expression	10, 20 mg/kg Mdivi-1 ↑ Functional recovery	Mdivi-1 protected neurons against cerebral ischemia via increased extracellular adenosine and CD39 expression	[40]
Male Sprague Dawley rats	Cardiac arrest model and restoration of spontaneous circulation (ROSC)	0.24 and 1.2 mg/kg Mdivi-1 After ROSC 1 min	After cardiac arrest ↑ brain Drp1 mitochondria ↓ brain Drp1 cytosol	-	1.2 mg/kg ↓ brain Cyto C ↓ Translocation of brain AIF ↓ brain caspase-3 activation	1.2 mg/kg ↑ Survival rate at 72 h ↑ OPC score at 72 h	Increased Drp1 after ROSC Inhibition of Drp1 had neuroprotective effects in cerebral ischemic injury	[44]
Male mice	30 min MCAO/23 h reperfusion	Deletion of mitochondrial A-kinase anchoring protein 1 (AKAP1)	Lacking AKAP1 ↑ Drp1 localization in mitochondria ↓ Drp1-S637 phosphorylation	Lacking AKAP1 ↑ Small mitochondria reduced contact site with ER ↑ Complex II dysfunction	Lacking AKAP1 ↑ Infarct volume ↑ Superoxide production Delayed calcium deregulation	⇿ Neurological deficit	A reduction of endogenous Drp1 inhibitor (AKAP1) increased stroke damage	[45]
Adult male Sprague Dawley rats	1, 3, 6, 12, 24 h pMCAO	1 mg/kg Mdivi-1 before pMCAO until end of experiment	-	↓ Selective mitophagy	↑ Infarct volume ↑ ROS ↑ Cyt-c release ↑ Caspase-3	↑ Neurological deficit	Drp1 inhibitor increased neurological deficit and infarct volume after pMCAO	[11]
Male Sprague Dawley rat	10 min global ischemia with sham IPC 10 min global ischemia with 2 min IPC	Mdivi-1 before onset of IPC and once daily until end of experiment	-	global ischemia with Drp-1 inhibitor ↓ COX4 and LC3	global ischemia with Drp-1 inhibitor ↓ Neurons in CA3 IPC with Drp-1 inhibitor ↓ Neurons in CA1 and CA3	-	Drp-1-mediated mitophagy pathway had neuroprotective effects against cerebral ischemia by removing mitochondrial dysfunction	[12]
Adult Sprague Dawley rats	90 min tMCAO	2.5-5.0 mg/kg p38 inhibitor 60 min before tMCAO	↓ Mitochondrial fission in ischemic cortex	↓ Mitophagy	↓ infarct size ↑ cell survival	↓ Neuronal deficit	p38 inhibition decreased mitochondrial fragmentation and mitophagy in cerebral I/R	[46]
Adult Sprague Dawley rats	90 min tMCAO/3 h reperfusion	Exercise (electronic treadmill) 2 weeks before tMCAO	↑ OPA1 ⇿ DRP1, MFF, Mfn1/2	↑ Mitochondrial function (COX II/III/IV)	↓ Brain edema	-	Exercise pretreatment increased mitochondrial fusion, decreased brain edema after cerebral I/R	[48]

AIF, apoptosis-inducing factor; AKAP-1, A-kinase anchoring protein 1; CREB, cAMP response element-binding protein; Cyt c, cytochrome C; Drp1, dynamin-related protein-1; ER, endoplasmic reticulum; IPC, ischemic preconditioning; I/R, ischemic/reperfusion; MAPK, major mitogen-activated protein kinase; OGD, oxygen-glucose deprivation; PKA, protein kinase A; tMCAO, transient middle cerebral artery occlusion; ROS, reactive oxygen species.

**Figure 4:**
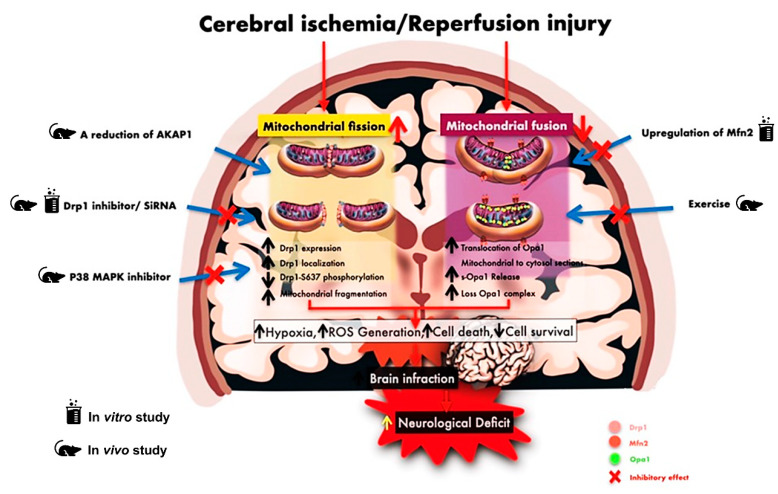
A summary of evidence in the interventions for the alterations in mitochondrial dynamics following ischemia/reperfusion: in vitro and in vivo studies. An increase in mitochondrial fission and a decrease in mitochondrial fusion were observed after cerebral ischemia/reperfusion. The upregulation of the Mfn2, exercise, inhibition of Drp1, and inhibition of P38 MAPK caused a decrease in mitochondrial fission, neuronal death, and neurological deficit. A reduction of AKAP1, which is an endogenous Drp1 inhibitor, increased mitochondrial fission, leading to an increase in brain infarct volume. AKAP-1, A-kinase anchoring protein 1; Drp1, dynamin-related protein-1; MAPK, major mitogen-activated protein kinases; Mfn2, mitofusin2; OPA1: optic atrophy protein1; s-OPA1, short optic atrophy protein1.

The conclusions from the in vivo studies still support the evidence that the application of a Drp1 inhibitor with Mdivi-1 after cerebral ischemia has the benefits of decreasing mitochondrial fission, neuronal death, neurological deficit, and improving the recovery of brain function. However, the results concerning the protective effect of a Drp1 inhibitor before and at the onset of reperfusion are still controversial, possibly due to the differences in study models in each investigation. Furthermore, the effects of modulating mitochondrial dynamics in cerebral ischemia/reperfusion have been associated with several conditions such as post cardiac arrest, AKAP1, P38MAPK, IPC, and exercise; therefore, further research in this area is essential. A summary of evidence in the interventions for the alterations in mitochondrial dynamics following ischemia/reperfusion in in vitro and in vivo studies is summarized in Figure 4.

## 8. Clinical Perspectives

A current therapeutic approach for cerebral ischemia can be via thrombolysis or reperfusion to restore the blood supply into the brain. However, rapid reperfusion can result in further injury to the brain area. Therefore, there is no neuroprotective therapy for cerebral ischemia with reperfusion, which has the benefit of reducing cerebral ischemia and improving neurological outcome. Several studies reported that imbalanced mitochondrial dynamics occurred after cerebral ischemia and ischemia/reperfusion [25,26,34,35,36,37,38,39,40]. Therefore, the mitochondrial-target approach and the interventions that improve or restore the balance of mitochondrial dynamics would be a potentially therapeutic strategy for cerebral ischemia to prevent further neurological damage. The mitochondrial fission inhibitor, the Drp-1 inhibitor, may be a valuable agent as an adjunctive treatment with the conventional therapy after cerebral ischemia from ischemic stroke or other neurological diseases such as traumatic brain injury with brain herniation, subarachnoid hemorrhage with vasospasm, or hypoxic-ischemic encephalopathy after a cardiac arrest condition. In addition, the Drp-1 inhibitor administrated before the onset of cerebral ischemia may have beneficial effects in the case of cerebrovascular diseases, including carotid occlusive disease, and moyamoya disease to prevent cerebral ischemia in the future. Therefore, further in vivo and clinical studies are still required to confirm the beneficial effects of modulating mitochondrial dynamics as an adjunctive therapy in the cerebral ischemic condition.

## 9. Conclusions

Changes in mitochondrial dynamics are important intracellular mechanisms occurring after cerebral ischemia/reperfusion. Of particular concern are the increased mitochondrial fission and decreased mitochondrial fusion. The imbalance of mitochondrial dynamics can lead to the induction of oxidative stress and neuronal death. Much of the evidence, from both in vivo and in vitro studies, shows beneficial effects as a result of modulating mitochondrial dynamics in oxygen glucose deprivation and cerebral ischemia/reperfusion. Therefore, the modulation of mitochondrial dynamics especially with the mitochondrial fission inhibitor could be a new therapeutic strategy in cases of ischemic stroke in the future. However, the mitochondrial dynamic changes with mitochondrial dynamic intervention need further study in clinical trials to confirm changes, models, and results of the treatment.

## Figures and Tables

**Table 1:** Evidence in the alterations in mitochondrial dynamics following oxygen-glucose deprivation/reoxygenation: in vitro studies.

Study Model	Injury Model	OGD			Reoxygenation			Interpretation	Reference
		Changes in Mitochondrial Dynamics	Mitochondrial Function/Morphology	Cell Survival	Changes in Mitochondrial Dynamics	Mitochondrial Function/Morphology	Cell Survival		
Mouse optic nerves	60 min OGD	↓ Drp-1 in cytosolic fraction	-	-	-	-	-	OGD-induced mitochondrial fission	[13]
HT22 murine hippocampal neuronal culture	45 min OGD/5 h reoxygenation	-	↑ Mitochondrial fragmentation	No activation of apoptosis	↑ s-OPA1	↑ Rapid and irreversible fragmentation	↑ Caspase 3/7 activation	An increase in rapid mitochondrial fragmentation and cell death were observed following OGD and OGD/R	[25]
Cortical and hippocampal neuronal culture from 18-day embryonic Sprague-Dawley rats	1 h OGD/24 h reoxygenation	-			Translocation OPA1 mitochondria to cytosol ↑ s-OPA1 (proteolytic process) Breakdown of OPA1 oligomer in mitochondria ↑ Mitochondrial fission	↑ Mitochondria fragmented and with granular appearance	↑ Cytochrome c release	OGD in neuronal cells induced mitochondrial fragmentation and cell death via alterations in OPA1	[33]
Primary astrocytic cultures	3 h hypoxia/10 h reoxygenation	↓ pDRP1 (ser637)	↓ mitochondrial area ↓ mitochondrial length ↓ VDAC ↑ LC3 II content ↓↓ ATP	↓ Astrocytic extensions ↓ Superoxide production	-	↓ mitochondrial area ↓ mitochondrial length ↓ number of mitochondria ↓ VDAC ↑ LC3 II content ↓ ATP	↓ Astrocytic extensions ↓ Superoxide production	Both hypoxia and reoxygenation caused mitochondrial fragmentation in primary astrocytes via increased mitochondrial fission	[31]
Primary cortical neuronal culture	3 h OGD/0.5-24 h reoxygenation	↓ Drp1, ↓ p-Drp1 (ser616) ↓ Mfn2 ⇿OPA1, Fis1	↓ normal mitochondria ↑ poorly labeled mitochondria ↑ swollen mitochondria	↑ apoptotic or necrotic signs	↓ Drp1, p-Drp1 (0.5–24 h of OGD/R) ↓ p-Drp1 (ser616) (3 h of OGD/R) ↑ Mfn1 (1–24 h of reoxygenation) ↓Mfn2 (0.5–24 h of OGD/R)	↑ VDAC (3-24 h of OGD/R) ↑ Complex V protein (1-24 h of OGD/R) ↑ mtDNA (12-24 h of OGD/R) ↑ numbers of large, swollen mitochondria with rupture/loss of internal cristae	↓ neuronal viability	OGD in neuronal cells induced mitochondrial fragmentation and cell death via the imbalance of mitochondrial fission and mitochondrial fusion	[32]

ATP, adenosine triphosphate; Drp1, dynamin-related protein-1; mtDNA, mitochondrial DNA; OGD, oxygen-glucose deprivation; OGD/R: oxygen-glucose deprivation/reoxygenation; OPA-1, optic atrophy protein1; s-OPA1: short isoform optic atrophy protein1; VDAC, voltage-dependent anion-selective channel 1.

**Table 2:** Evidence in the alterations in mitochondrial dynamics following cerebral ischemia/reperfusion injury: in vivo studies.

Study Model	Injury Model	Ischemia			Reperfusion			Interpretation	Reference
		Changes in Mitochondrial Dynamics	Mitochondrial Function/Morphology	Cell Survival	Changes in Mitochondrial Dynamics	Mitochondrial Function/Morphology	Cell Survival		
C576BL/6 Mice	12, 24 h MCAO	Ischemic hypoxic condition ↓ Mfn2 (12, 24 h)	-	-	-	-	-	Decreased Mfn2 expression in ischemic and hypoxic conditions	[38]
Male Sprague-Dawley rats	8 min global cerebral ischemia/4,6,24 h reperfusion	-	-	-	↑ s-OPA1 release Loss OPA1 complex	↑ Mitochondrial fragmentation Loss of normal cristae structure in brain mitochondria	↑ Cyt c release ↑ Caspase 3/7 activation	Decreased mitochondrial fusion during global brain ischemia	[25]
Adult male Wistar rats	15 min global brain ischemia/1, 3, 24, 72 h reperfusion	↑ Mfn2 translocation to cytoplasm during global brain ischemia	-	-	↓↓ Mfn2 in cortical mitochondria ↓ Mfn2 translocation to cytoplasm during reperfusion	-	↑↑ VDAC1 in hippocampus following 3 and 72 h of reperfusion	Increased Mfn2 release from mitochondria during early reperfusion associated with mitochondrial and neuronal dysfunction	[35]
C576BL/6 Mice	2 h MCAO/3 h Reperfusion	-	-	-	-	↑ Small, spherical mitochondria	-	Increased mitochondrial fission in early phase of ischemic stroke	[36]
8-week old male mice	90 min transient MCAO/2, 7, 14, 28 days reperfusion	-	-	-	Ischemic core ↓ Drp1 ↓ OPA1 Ischemic penumbra ↑ OPA1 ↑ Drp1 ↑ pDrp1	-	-	Mitochondrial fission and fusion were decreased in ischemic core but increased in ischemic penumbra area	[27]
Male transgenic mice with mitochondrial tagging	10 min global brain ischemia/2, 4, 24 h and 3 day reperfusion	-	-	-	↑ Mitochondrial fission(2hr) ↑ Mitochondrial fusion (started after 24 h to D3)	↑ Mitochondrial fragmentation during 24 h recovery	-	Resistantischemic neurons can shift the mitochondrial fission to fusion process after 24 h of ischemia.	[37]
Adult male Sprague Dawley rats	90 min transient MCAO/6, 14, or 22 h reperfusion	-	-	-	At 14,22 h ↑ Drp1		At 22 h ↑ LC3-II/I ↑ Parkin translocation	Mitochondrial recruitment of Drp1 after 22 h of I/R via Parkin-mediated mitophagy.	[38]

Cyt c, cytochrome C; Drp1, dynamin-related protein-1; pDrp1, phosphorylated dynamin-related protein-1; MCAO, middle cerebral artery occlusion; Mfn2, mitofusin2; OPA-1, optic atrophy protein1; s-OPA1, short isoform optic atrophy protein1; I/R, ischemic/reperfusion; VDAC1, voltage-dependent anion channel-1.

**Table 3:** Evidence in the interventions for the alterations in mitochondrial dynamics following oxygen-glucose deprivation/reoxygenation: in vitro studies.

Study Model	Injury Model	Intervention	Major Findings				
			Changes in Mitochondrial Dynamics	Mitochondrial Function/Morphology	Cell Survival	Interpretation	Reference
HT-22 hippocampal cells	3- and 5-mM Glutamate -induced cell death	50, 75 µM Mdivi-1/18 h after glutamate induced toxicity 10 min SiRNA of Drp1prior to glutamate induced toxicity	Drp1 siRNA and Mdivi-1 ↓Fragmentation	Drp1 siRNA and Mdivi-1 ↓ Depolarization ↓ Mitochondrial membrane potential (MMP)	Drp1 siRNA and Mdivi-1 ↓ Apoptotic cells	Inhibition of mitochondrial fission attenuated neural death against glutamate toxicity	[8]
Primary rat embryonic cortical neurons	2-mM Glutamate induced cell death 4 h OGD/18–24 h re-oxygenation	25 µM Mdivi-1 18 h after OGD and glutamate induced neuronal toxicity	-	-	↓ Apoptotic cells	Inhibition of mitochondrial fission attenuated neural death in OGD and glutamate induced neurotoxicity.	[8]
HT-22 hippocampal cells	5 mM Glutamate-induced cell death	72 h SiRNA Drp1 prior to glutamate induction	-	↓ Mitochondrial swelling ↑ ATP production ↓ MMP ↑ Calcium buffering capacity	↓ LDH release ↓ Bax/Bcl-2 ratio ↓ Cleavage of caspase 9/3 ↓ Cyto C release	Drp1 siRNA protected HT22 cells from glutamate mediated excitotoxicity and preserved mitochondrial function via regulation of intracellular calcium homeostasis.	[28]
SH-SY-5Y neuroblastoma cells	90 min OGD/24 h reoxygenation	10 µM Mdivi-1 5 min before OGD Drp1 siRNA 48 h before OGD		Knockdown or inhibition of Drp-1 ↓ Mitochondrial morphology change ↑ Mitochondrial function	Knockdown or inhibition of Drp-1 ↓ Cell death ↓ Cyto C release in cytosol ↓ Bax in mitochondrial fraction	Drp1 inhibition protected neurons against OGD via maintenance of mitochondrial function and morphology and decreased Bax oligomerization in mitochondria	[9]
Primary hippocampal neurons	6 h OGD/20 h reoxygenation	50 µM Mdivi-1 40 min before OGD	-	-	↓ ROS ↑ SOD activity ↓ MDA level ↓ Apoptosis cell ↑ Bcl-2 expression ↓ Cyto C release	Mdivi-1 protected hippocampal neurons from OGD induced apoptosis by suppressing ROS-mitochondrial pathway	[39]
Primary astrocyte culture	6 h OGD	5, 10, 20, 30 µM Mdivi-1 0, 2, 6, 12, 24,48 h siRNA of CD39 before OGD	-	-	Mdivi-1 (20 µM) ↑↑ CD39 expression (at 1 h) ↑ AMP (2 h) ↑↑ pCREB (2 h) ↑↑ PKA (1 h) siRNA of CD39 ↑ ADP ↓ AMP	Mdivi-1 increased ATP metabolism by upregulation of CD39 expression via cAMP/PKA/CREB signaling pathway CD39 had the ability to degrade primary astrocytic ADP to AMP	[40]
SH-SY-5Y neuroblastoma cells	6 h OGD/1 h reoxygenation	10 µM Mdivi-1 30 min before OGD 10 µM, 10 mM Β-hydroxybutyrate 30 min before OGD	Mdivi-1 and Β-hydroxybutyrate ↓ cell with aggregated Drp1	Mdivi-1 and Β-hydroxybutyrate ↓ Percentage of fragmented mitochondria ↓ the decrease in mitochondrial membrane potential	Mdivi-1 and Β-hydroxybutyrate ↑ Cell viability ↓ LDH ↑ ATP ↓ ROS	Mdivi-1 and Β-hydroxybutyrate increased cell viability from OGD by improved mitochondrial dynamic and mitochondrial function	[42]
Mouse N2a neuroblastoma cells	4 h OGD/4 and 12 h reoxygenation	Parkin overexpression Drp1 knockdown	Parkin overexpression ↑ Drp1 degradation	Parkin overexpression ↓ Mitochondrial dysfunction	Parkin overexpression ↓ Apoptosis Drp1 knockdown ↓ Mitochondrial damage and apoptosis	Parkin protected mitochondrial dysfunction and apoptosis after OGDR	[30]
PC 12 cells	48 h OGD	Drp1 siRNA (prior to OGD)	-	↑ LC3-II accumulation	-	Mitochondrial fission required for selective mitochondrial autophagy under OGD conditions	[11]
Primary hippocampal neurons	3 h OGD/24 h reoxygenation	Drp1 siRNA (48 h before OGD)	-	-	Drp-1 siRNA ↑ LDH release ↑ Release Cyt c ↑ ROS	Drp-1 suppression was associated with neuronal damage in primary hippocampal neurons	[12]
HT-22 hippocampal neuron cells	100, 200, 300, 400 and 500 µM CoCl2/24 h	Down-/up-regulation of Mfn2	-	Upregulation of Mfn2 ↓ Hypoxia induced loss of MMP	Upregulation of Mfn2 ↓ Apoptosis ↓ Phospho-ERK Downregulation of Mfn2 ↑ Apoptosis	Mfn2 overexpression attenuated hypoxia-induced apoptosis via the mitochondrial apoptotic pathway	[34]

AMP, adenosine monophosphate; ADP, adenosine diphosphate; Bax, Bcl-2-assocaited X protein; Bcl2, B-cell lymphoma2; CREB, cAMP response element binding protein; Cyt c, cytochrome C; Drp1, dynamin-related protein-1; LC3-II, microtubule-associated protein-II; LDH, lactate dehydrogenase; MDA, malondialdehyde; MMP, mitochondrial membrane potential; OGD, oxygen-glucose deprivation; OGDR, oxygen-glucose deprivation/reoxygenation; pCREB, Phospho-CREB; PKA, protein kinase A; Drp1 siRNA, silencing of Drp1; ROS, reactive oxygen species; SOD, superoxide dismutase.

## Abbreviations

AIF	Apoptosis-inducing factor
AKAP-1	A-kinase anchoring protein-1
ATP	Adenosine triphosphate
Bax	Bcl-2-assocaited X protein
Bcl-2	B-cell lymphoma2
Cyt c	Cytochrome C
Drp1	Dynamin-related protein-1
Drp1 siRNA	Silencing of dynamin-related protein-1
p-Drp1	Phosphorylated dynamin-related protein-1
IPC	Ischemic preconditioning
LC3-II	Microtubule-associated protein-II
MAPK	Major mitogen-activated protein kinase
MCAO	Middle cerebral artery occlusion
tMCAO	Transient middle cerebral artery occlusion
MDA	Malondialdehyde
Mfn2	Mitofusin 2
mtDNA	Mitochondrial DNA
OGD	Oxygen-glucose deprivation
OGDR	Oxygen-glucose deprivation/reoxygenation
OPA1	Optic atrophy protein1
s-OPA1	Short isoform optic atrophy protein1
PKA	Protein kinase A
ROS	Reactive oxygen species
SOD	Superoxide dismutase
VDAC-1	Voltage-dependent anion-selective channel 1
